# THE UNEVEN LATER WORK COURSE: INTERSECTIONAL GENDER, AGE, RACE, AND CLASS DISPARITIES

**DOI:** 10.1093/geroni/igad104.0440

**Published:** 2023-12-21

**Authors:** Phyllis Moen, Sarah Flood, Janet Wang

**Affiliations:** University of Minnesota, Minneapolis, Minnesota, United States; University of Minnesota, Minneapolis, Minnesota, United States; University of Michigan, Ann Arbor, Michigan, United States

## Abstract

We document patterned continuity and change in monthly work attachments and analyze the intersecting effects of age, gender, education, and race/ethnicity. We utilize 16-month panel data from Current Population Survey (2008-2016) to code monthly states: working full-time, long hours, part-time; being self-employed or unemployed; not working due to disability or family care, or retired. Analyses of 346,488 American women and men aged 50–75 years reveal patterned elasticity in the timing and nature of work attachments in the form of six distinctive pathways. Analyses illustrate divergences and disparities: advantages for educated White men, disadvantages for low-educated Black men and women through their early 60s, and intersecting effects of gender, education, and race/ethnicity during the later work course across age groups. We find convergence across social markers by the 70s. This research highlights the importance of intersectional analysis, recasting the gendered work course in later adulthood to reflect its complexities.

